# Molecular Drivers of Developmental Arrest in the Human Preimplantation Embryo: A Systematic Review and Critical Analysis Leading to Mapping Future Research

**DOI:** 10.3390/ijms22158353

**Published:** 2021-08-03

**Authors:** Konstantinos Sfakianoudis, Evangelos Maziotis, Eleni Karantzali, Georgia Kokkini, Sokratis Grigoriadis, Amelia Pantou, Polina Giannelou, Konstantina Petroutsou, Christina Markomichali, Maria Fakiridou, Michael Koutsilieris, Byron Asimakopoulos, Konstantinos Pantos, Mara Simopoulou

**Affiliations:** 1 Centre for Human Reproduction, Genesis Athens Clinic, 14–16 Papanikoli, 15232 Athens, Greece; sfakianosc@yahoo.gr (K.S.); lina.giannelou@gmail.com (P.G.); dinapetroutsou@hotmail.com (K.P.); chrimarko@hotmail.com (C.M.); maria_fkrd@hotmail.com (M.F.); info@pantos.gr (K.P.); 2 Department of Physiology, Medical School, National and Kapodistrian University of Athens, 75 Mikras Asias, 11527 Athens, Greece; vagmaziotis@gmail.com (E.M.); elenikarantzali@gmail.com (E.K.); ginakokini@gmail.com (G.K.); sokratis-grigoriadis@hotmail.com (S.G.); mkoutsil@med.uoa.gr (M.K.); 3 Department of Physiology, Medical School, Democritus University of Thrace, 68100 Alexandroupolis, Greece; basima@med.duth.gr; 4 Genesis Genoma Lab, Genetic Diagnosis, Clinical Genetics & Research, 15232 Athens, Greece; amelia.pantou@gmail.com

**Keywords:** embryo arrested development, IVF, infertility etiologies

## Abstract

Developmental arrest of the preimplantation embryo is a multifactorial condition, characterized by lack of cellular division for at least 24 hours, hindering the in vitro fertilization cycle outcome. This systematic review aims to present the molecular drivers of developmental arrest, focusing on embryonic and parental factors. A systematic search in PubMed/Medline, Embase and Cochrane-Central-Database was performed in January 2021. A total of 76 studies were included. The identified embryonic factors associated with arrest included gene variations, mitochondrial DNA copy number, methylation patterns, chromosomal abnormalities, metabolic profile and morphological features. Parental factors included, gene variation, protein expression levels and infertility etiology. A valuable conclusion emerging through critical analysis indicated that genetic origins of developmental arrest analyzed from the perspective of parental infertility etiology and the embryo itself, share common ground. This is a unique and long-overdue contribution to literature that for the first time presents an all-inclusive methodological report on the molecular drivers leading to preimplantation embryos’ arrested development. The variety and heterogeneity of developmental arrest drivers, along with their inevitable intertwining relationships does not allow for prioritization on the factors playing a more definitive role in arrested development. This systematic review provides the basis for further research in the field.

## 1. Introduction

In vitro fertilization (IVF) is rapidly evolving, with research focusing on improving the probabilities of a live-birth. The focal point of IVF research is the developmental dynamic of the preimplantation embryo, which is clinically cultured in vitro for a maximum of 5–7 days [1]. Only the embryo(s) of the highest developmental potential are selected for transfer. To assess the developmental potential of the embryo, a number of models assessing morphology, morphokinetics and numerous biomarkers have been proposed [2,3]. Arrested embryo development is characterized by downregulation of metabolic activity and cessation of cell division [4]. In order for an embryo to be characterized as non-viable—or arrested—it should present with a lack of cleaving activity and fail to show a sign of mitotic cellular division for at least 24 hours. Additionally, blastomeres of arrested embryos may have undergone full degeneration or lysis [5]. Developmental process irregularities may include slow developing embryos. These embryos may proceed to become arrested, or they might continue to develop and, in some cases, reach mature blastocysts even leading to a live-birth with good neonatal outcomes-albeit prognosis of slow developing embryos is poor. Slow developing embryos constitute a separate category featuring cleavage irregularities and should not be discarded prior to the 24-hour period of confirmed ceased development [6,7]. A 60% blastocyst formation benchmark value is reported by the ESHRE Key Performance Indicators. Hence, it is anticipated that 40% of the total cohort of zygotes arrest prior to reaching blastocyst stage [8]. Along with the data that over 40% of patients present with at least one arrested embryo in each IVF cycle [6,9], this information may be viewed as reporting on prevalence of the phenomenon on arrested development. Embryo arrest may occur on different developmental stages, marking the different milestones that a preimplantation embryo is expected to reach during the 6 days culture in the IVF laboratory. Depending on the developmental stage that embryo arrest is noted one can observe failure of a zygote to cleave, cleavage stage arrest either on day 2 or day 3 when the embryo is expected to be between the 2 and 8-cell stage, failure to compact to morula, or failure to form a blastocyst. 

Various mechanisms leading to arrested development have been proposed coupled by numerous explanations on why these mechanisms may be instigated [9]. One of the most prominent theories views arrest as a protective mechanism for averting further development of poor-quality embryos [9,10,11]. Another hypothesis suggests that other than developmental arrest reflecting a predestined intrinsic scenario of natural selection processes where poor-quality embryos are “cut-off”, we should also consider the possibility that it is the demanding nature of reaching these milestones itself coupled by the respective in vitro culture laboratory conditions that play an important role in failure of embryos to reach these milestones and arrest [12].

Studying, understanding and predicting developmental arrest is at the spotlight of research. It has been established that the number of available embryos for transfer is positively associated with live-birth rate [13]. Thus, assessing factors leading to embryonic arrest will enable understanding and addressing of the phenomenon, ultimately resulting in enhancing IVF outcomes. Recently, the introduction of time lapse monitoring (TLM) has provided an additional tool allowing continuous observation of embryos during culture, enabling morphokinetic data to contribute to the embryo selection process [14,15]. Despite the widespread use of morphokinetic analysis lately, it has been reported that this method failed to shed light on the subject of early embryonic arrest [16]. When examining the efficiency of morphology grading, morphokinetics and omics in predicting developmental advancement it appears that none can be individually considered to be entirely reliable in predicting developmental advancement [2]. Trying to dissect the multifactorial event that arrested development is, one would expect to find genetic abnormalities in the core of it. Indeed, chromosomal abnormalities such as aneuploidy, polyploidy and mosaicism represent the major cause of developmental arrest [6,17,18]. Various studies have demonstrated a relationship between aberrant cleavage patterns and cell death due to genomic loss [16,19]. Chromosome loss due to mitotic errors does not reveal a propensity of maternal or paternal source [6,20]. The fact that preimplantation embryos are highly susceptible to cell division errors renders the developmental process more fragile than robust [21]. What data has indicated is that delineating the molecular pathways entailed in developmental advancement represents the holy grail in the search for the cause of developmental arrest. Investigating this complex phenomenon from a molecular perspective allows for studying the complex relationships between the different levels of interaction leading to this abnormality that dictates embryonic fate. There is evidence supporting that several molecular pathways such as WNT MAPK pathway may be implicated in embryonic developmental control [22]. A failure of crucial gene activation especially between the 4- and 8-cell stages, or a single gene mutation in genes that are key to the developmental process, may lead consequently to cleavage arrest [23]. Moreover, reactive oxygen species (ROS), inducing oxidative stress have been associated with embryonic arrest [24], while, a number of parental factors may hinder developmental potential of embryos. It has been observed that paternal [25] and maternal age [26], as well as male ([27]) and female factor infertility etiologies [28,29] have been associated with early embryonic arrest. 

Embryo developmental arrest is a multifactorial phenomenon subject to intrinsic and extrinsic parameters. Investigating the causative relationships ruling the developmental arrest event, it appears that the list of the usual suspects includes maternal or paternal factors, embryonic metabolism activity, impairment of crucial molecular pathways that ceases the developmental process and genetic abnormalities, or laboratory protocols and conditions that may serve as culprits. Surely, extrinsic factors may equally lead to developmental arrest through, trauma, stress, of the said gametes and embryos as data has shown. These extrinsic factors may be described as techniques employed in the IVF laboratory that gametes and embryos are subjected to, along with conditions of embryo culture This constitutes an equally interesting area of research. Two comprehensive reviews regarding developmental arrest and one systematic review focused on the embryonic abnormal developmental patterns have been recently published [30,31,32], indicating the explosion of interest noted on the subject at hand. The present systematic review uniquely provides to literature an original and in-depth critical analysis on available data reporting strictly on intrinsic factors associated with developmental arrest in human embryos and investigating molecular mechanisms and pathways driving developmental arrest. The study focuses on investigating the underlying mechanisms along with the molecular pathways that shape and guide the developmental arrest process. The wealth and volume of data on the subject of developmental arrest and concurrently the lack of a conclusive, all-inclusive report on the factors driving developmental arrest related to the embryo’s and its parents’ molecular blueprint has served as the driver to conduct this timely and essential study. The phenomenon of developmental arrest generates two main pillars of research. The first pertains to investigating means and routes to avoid developmental arrest. The second pillar focuses on research indicating how we can predict developmental arrest in a timely fashion that could allow us to manage respective embryos accordingly enabling better decision-making in the IVF-laboratory. The authors aim to present current knowledge and hypotheses, identify areas that merit further investigation and map future research. Information provided herein might assist in formulating novel approaches to increase the possibilities for a successful IVF cycle.

## 2. Materials and Methods

**Figure 1:**
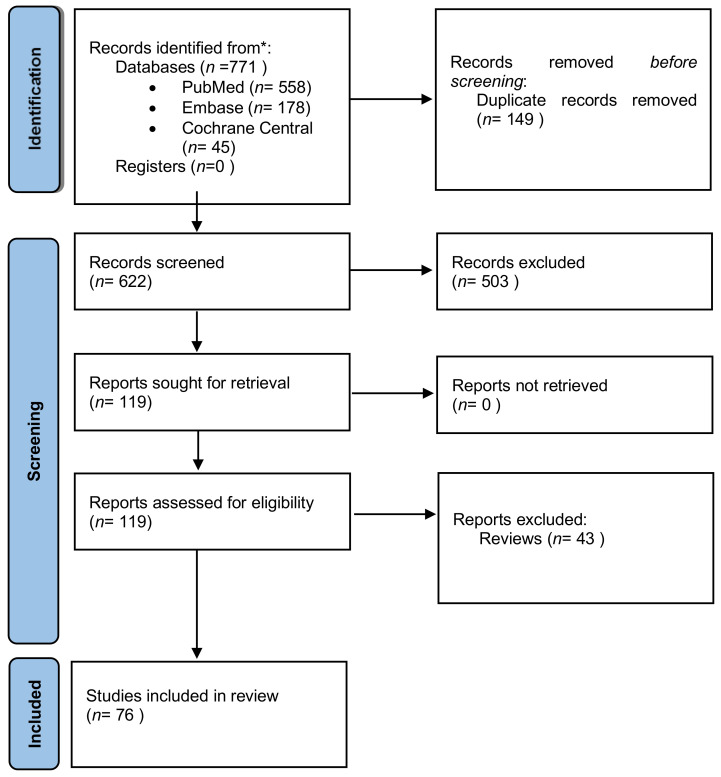
PRISMA flowchart regarding study selection.

A systematic search on PubMed/Medline, Embase and Cochrane Central Database was performed on 22 January 2021. The keywords employed and combined for the search strategy were “in vitro fertilization”, “IVF”, “assisted reproduction”, “intracytoplasmic sperm injection”, “ICSI”, “embryo arrest”, “embryonic arrest”, “arrested development” and “developmental arrest”. The original search yielded 771 studies from the three databases. Following removal of duplicate studies (*n* = 149), all records were screened and full-text was sought and obtained for relevant articles. Relevant articles (*n* = 119) were identified following title and abstract screening, employing the flow chart of Preferred Reporting Items for Systematic Reviews and Meta-analysis (PRISMA) as presented in Figure 1. Screening and selection of literature was performed independently by three authors (EM, EK and GK). Disagreements between the authors were resolved by an arbitration mediated by the senior authors (KP and MS). Backward and forward citation mining employing Google Scholar was performed. This systematic review reports only on studies on human embryos. The search was limited to full-length manuscripts published in English in peer-reviewed journals up to 22 January 2021. A total of 76 studies were found eligible to be included in the present systematic review. All 76 relevant studies are included and thoroughly described following the strict methodology of a systematic review. It should be underlined that this study is not strictly limited in the traditional design of the systematic review, but rather aims beyond that. To elaborate on this, with the systematic review serving as the basis the authors concurrently performed an all-inclusive critical analysis that explores the factors and pathways hindering embryo developmental potential, providing direction for future research and identifying areas of interest.

## 3. Results

With regards to the results’ analysis, reporting on associations and drivers of arrested development of the preimplantation embryo, the authors decided that studies should be categorized in two major groups and presented accordingly. Firstly, this study analyzes intrinsic factors related to the embryo’s identify and profile that lead to developmental arrest. These include aspects entailed in the embryo’s cellular and molecular behavior namely pertaining to gene expression, mitochondrial DNA, methylation patterns, small non-coding RNAs, chromosomal abnormalities, embryonic metabolism and vacuolization which has been thoroughly documented to be associated with arrested development. Secondly, this work focuses on showcasing maternal as well as paternal factors that data has shown to be associated to arrested development and have been suggested to predispose embryos to developmental arrest.

### 3.1. Intrinsic Embryonic Factors Leading to Arrest

A total of 34 studies were identified evaluating embryonic factors leading to developmental arrest. The factors indicated as developmental arrest drivers were differential gene expression (*n* = 6), mitochondrial DNA (*n* = 6), methylation patterns (*n* = 3), small noncoding RNAs (*n* = 1), chromosomal abnormalities (*n* = 7), embryonic metabolic profile (*n* = 9), while specific morphological characteristics (*n* = 2), namely vacuolization were found to be associated with developmental arrest.

#### 3.1.1. The Role of Embryonic Gene Variation and Expression

A number of different genes with an essential role in pre-implantation development participating in crucial processes and molecular pathways have been reported to be implicated in developmental arrest [33,34]. Non-viable embryos express different patterns of genes compared to viable embryos. Essential processes namely mitotic cell division, oocyte maturation, chromosome segregation, cell cycle checkpoint control and ploidy regulation seem to be impaired in arrested embryos. Furthermore, misexpression of genes accountable for chromosomal epigenetic modification and gene transcription such as DNA-methyltransferase 3 beta (*DNMT3B*), Histone Deacetylase 1 (*HDAC1*), Tet Methylcytosine Dioxygenase 3 (*TET3*) and Yin Yang 1 (*YY1*) may lead to higher rates of embryonic loss [34]. Pre-implantation arrest may be also caused by insufficient expression of genes including Autophagy Related 5 (*ATG5*), Cullin 3 (*CUL3*), Ubiquitin Specific Peptidase 11 (*USP11*) and Ubiquitin Specific Peptidase 2 (*USP2*), all instrumental in protein synthesis, ubiquitination and autophagy [34].

Extensive DNA damage may be responsible for developmental arrest. Non-viable embryos express lower levels of genes which play a key role in DNA repair and telomere maintenance mechanisms including Breast Cancer 1 (*BRCA1*), Telomeric Repeat Binding Factor 1 (*TERF1*), ERCC Excision Repair 1 (*ERCC1*), Endonuclease Non-Catalytic Subunit (*ERCC1*), X-Ray Repair Cross Complementing 6 (*XRCC6*) [34]. Comparing viable and arrested embryos revealed a misexpression of genes involved in fertilization, such as Inositol 1,4,5-Trisphosphate Receptor Type 1 (*ITPR1*), a main receptor for initiating the Ca2+ oscillations that could damage cortical granule release and zona-hardening [34]. In the attempt to investigate a possible correlation between specific genes and embryo quality another study revealed that the expression of several genes is related to the morphokinetic parameters of the embryo [33]. Bone morphogenetic protein 15 (*BMP15*), which is highly connected with the function of granulosa and theca cells plays a vital role in follicular growth and fertilization [35,36,37,38]. It is responsible for the decreased activity of connexin Cx43 in granulosa cells [39]. Studies indicated that the expression of *BMP-15* was significantly higher and Cx43 significantly lower in normally developing embryos than in arrested [33]. The specific pattern of expression with Cx43 and high levels of *BMP-15* mRNA has been suggested as an indicator of embryo competence [33].

It should be noted that data pertaining to the role of embryonic gene variation and expression in arrested development largely requires confirmation and retains an experimental status. However, examination of these genes in a clinical setting would be challenging as preimplantation genetic testing (PGT) may be required in order for the data acquired to hold any prognostic value. This fact poses two major concerns. Firstly, PGT being an invasive procedure would add another level of complexity that ultimately may compromise the embryo itself and one would have to weigh benefit versus detriment. Secondly, PGT is commonly performed on blastocysts, thus the embryos that present with differential expression levels will have already arrested failing to reach the biopsiable stage. It may be of importance to further investigate factors that may influence expression levels. These factors may range from maternal or paternal mutations on gene promoters or silencers to epigenetic modifications due to laboratory conditions. Hence, the intertwined nature of the relationships between the factors leading to developmental arrest is highlighted.

#### 3.1.2. The Role of Mitochondrial DNA

A novel indicator to enhance identification and selection of embryos with the highest blastulation potential is mitochondrial DNA (mtDNA), evaluated in the culture medium [40,41,42]. A strong correlation between mtDNA/gDNA (genomic DNA) ratio and embryo fragmentation has been observed reflecting formation of anucleate cytoplasmic fragments which release gDNA [41]. Specifically, Day-3 embryos that form a blastocyst, secrete significantly higher levels of mtDNA into media than those arresting [41]. Moreover, a higher mtDNA/gDNA ratio was observed on Day-3 in embryos successfully reaching blastocyst stage compared with the arrested group [41]. When evaluating mtDNA via biopsy another study reported that mtDNA/nDNA ratio (nuclear DNA), known also as mitochondrial content (MC), was significantly higher in arrested embryos and in day 6 aneuploid blastocysts compared to euploid blastocysts [40]. This may be explained by defects in cell division and degradation of nDNA [40]. Moreover, the fact that differences in MC are not observed prior to Day-6, disputes the theory that an increase in mitochondrial copy number is a response to a stressful condition [40]. Generally, mtDNA mutations may prevent the continuous mitochondrial metabolic activity that is required for the maturation of the oocytes [43,44,45]. A study has reported that frequencies of 4977-bp mtDNA deletion -the most common mtDNA deletion- were almost double in unfertilized oocytes than in arrested embryos [45,46]. Several fundamental structural genes including ATP synthase subunit 6 (ATPase 6) and 8 (ATPase 8), cytochrome oxidase III (*COXIII*), and NADH-CoQ oxidoreductase were differentially expressed due to mtDNA 4977-bp deletion [45]. This deletion may prove harmful for gene expression [45]. Additionally, a downregulation of cytochrome c oxidase subunit II (*COXII*) and ATP synthase subunit 6 (*MTATP6*) expression levels in cumulus cells (CCs) was detected in non-viable embryos, unveiling the importance of proper mitochondrial function at the earliest stages of development [33]. A mitochondrial dysfunction could be indicated also from the increased expression of heme oxygenase-1 (*HO-1*) gene in arrested embryos as an extensive oxidative stress may be harmful for oocyte competence [33,47]. A negative relationship between mRNA levels of the genes and the assessed morphokinetic parameters was noted with a lower expression of *BMP15*, *COXII*, and *MT-ATP6* observed in arrested embryos [33].

Mitochondrial DNA copy number has been explored as an option of a non-invasive biomarker on embryo competence. Although a number of studies have reported a correlation between mtDNA and IVF cycle outcome, there is controversy regarding the efficacy of mtDNA quantitation as a beneficial adjunct to embryo selection. Other studies suggest that mtDNA is a negligible predictor of implantation rates [48,49,50]. Moreover, no association of mtDNA content with blastocyst ploidy and viability has been reported [51]. It has been proposed that these heterogenous results may be attributed to the difference in the setting of the study. The majority of studies that reported lack of correlation between mtDNA and embryo competence, were conducted on a single clinic. Despite the fact that single-center studies are considered to be of lesser impact compared to multi-center studies, this design enables enhanced verification of the validity of findings, as numerous potential laboratory variables including culture media and biopsy technique are better controlled [51]. However, data is still limited thus safe conclusions should not be extrapolated. It should be noted that investigations on the role of mitochondrial DNA on arrested development has led to discrepant results and this data has yet to be validated before it is considered accepted knowledge. Our goal to control, predict, or avoid arrested development may be noble, and employment on mtDNA copy numbers appears as a promising option that merits investigation. Nonetheless, the controversy surrounding its use has been thoroughly documented [48,52]. Conduction of further well-designed studies is required, prior to employing this biomarker in a clinical setting.

#### 3.1.3. The Role of Methylation Patterns

During human development both paternal and maternal genome activation is required [53]. Consequently, genes with specific parent-of-origin expression defined as ‘imprinted genes’ are required for proper embryo development. They are controlled from differentially methylated specific DNA sequences called imprinting control regions (ICRs) [54]. There are two waves of genome-wide demethylation with the first one occurring during germ cells’ development and the second one post-fertilization [55,56]. H19DMR is one of the ICRs involved with the expression of two imprinted genes: H19 expressed from the maternal allele and insulin growth factor 2 (IGF2) expressed from the paternal allele [57]. A higher number of arrested preimplantation embryos present with abnormal imprinting at H19 on both paternal and maternal alleles in contrast with suitable for transfer blastocysts [58]. In particular, more than half of the arrested embryos exhibited a significant hypomethylation of the paternal allele independently of developmental stage [58]. It is believed that this is attributed to an instability of the methylation maintenance in the early developmental stages as the methylation process during gametogenesis was reported to occur normally [58]. On the other hand, hypomethylation of the maternal allele observed in some embryos is hypothesized to be attributed to an abnormal erasing of the paternal imprint in the maternal germ line, thus affecting the monoallelic expression [58]. In addition, another study evaluated the methylation profile of two transcription factors, Nanog and octamer-binding transcription factor 4 (Oct4), both crucial for the establishment of pluripotency during embryonic development [59,60,61,62,63]. The majority of the arrested embryos exhibited a significant hypermethylation of the Oct4 promoter while the Nanog promoter remained unmethylated [59]. Paternal allele seemed to be resistant to demethylation, as higher levels of Oct4 hypermethylation were observed in embryos from couples presenting with male factor infertility. However, further investigation is required to clarify if this hypermethylation is associated with a specific cause of infertility [59].

Methylation patterns are an epigenetic modification regulating gene expression. Epigenetic modifications may be mainly attributed to environmental factors. These factors in the case of the preimplantation embryos are mainly culture conditions and techniques employed in the IVF laboratory. It should be noted that the role of methylation patterns in decoding arrested development has still a long way to go before formed hypotheses become widely accepted knowledge and a true “ally” in deciphering what leads to arrested development. Further studies should be designed to concur on the value of reporting on the methylome profile of the embryo in the context of the IVF laboratory as a predictive tool. Such a tool would enable identification of the actual factors that impact the methylome compromising the embryo.

#### 3.1.4. The Role of Small Noncoding RNA

Only one study evaluated the expression of small noncoding RNAs (sncRNA) in preimplantation embryos [64]. It was observed that arrested embryos presented with a differential expression of 13 sncRNAs when compared with good quality blastocysts. It should be noted that the evaluation was performed by examining spent culture media. Eleven out of 13 were Piwi-interacting RNAs (piRNA) and the remaining two were microRNAs (miRNA) of the let-7 family -namely, let-7i-5p and let-7b-5p. These sncRNAs target genes encoding transcriptional regulators, metabolite interconversion enzymes, binding proteins and their activity modulators, transmembrane signal receptors, scaffold proteins and transporter proteins. However, only one of these sncRNAs, namely the miRNA hsa-let-7i-5p was observed to be differentially expressed when compared to both high and low-quality blastocysts. miRNAs along with piRNAs have emerged as factors that merit further investigation as they affect multiple layers of molecular pathways entailed in biological processes. Their potential in investigating the pathophysiology of infertility in general from endometriosis [65] to diminished ovarian reserve [66] has been documented. It is undoubtable that they constitute an area of interest for future research extending to embryo developmental arrest. Since only a single study has been published on the role of small non-coding RNAs in developmental arrest, albeit it may represent an area of interest that merits exploring, undoubtably on this experimental data further research is required to validate the observations.

#### 3.1.5. The Role of Chromosomal Abnormalities

Chromosomal abnormalities are a common observation in pre-implantation embryos. Studies based on FISH technique observed that a high number of embryos presented with mosaicism and a significant correlation between aneuploidy and developmental arrest [67,68,69,70,71,72]. However, a recent study, based on whole genome sequencing techniques, and employing over 35,000 embryos, observed that aneuploidy is not associated with developmental arrest until the blastocyst stage. The study observed that aneuploid embryos presented with a significantly slower development [73]. On the other hand, a recent review proposed that several molecular signaling pathways, namely Wnt, MAPK and Hippo pathways may be involved in embryonic aneuploidy and lead to developmental arrest and/or implantation failure [22].

A higher rate of nuclei with apoptotic chromatin has been observed in blastomeres of embryos of poor morphology and with extensive fragmentation compared to embryos with good morphology [74], while a high rate of embryos with significant degree of fragmentation have been reported to be euploid [74]. Interestingly, reciprocal chromosomal translocations referring to whole chromosomes between blastomeres have been observed in embryos with micronucleation [75]. It has been voiced that arrested and slow-developing embryos may express a higher rate of abnormalities [76]. Multinucleation and mosaicism may lead to a delayed timing of cleavage and extensive fragmentation [77,78,79]. Any deviation from the optimal time-frame of reaching each cleavage stage may be detrimental for embryonic development [76,80,81]. Moreover, it has been observed that abnormal cleavage pattern may lead to arrest [76].

Segmental aneuploidy is an outcome of DNA double stranded breaks (DSBs) derived from various factors such as oxidative stress [82]. An interruption of DNA synthesis may result to a double-strand break. Segmental aneuploidies, for the majority of cases, are of mitotic origin, in contrast to whole chromosomal abnormalities which are principally of meiotic origin. In a recent study a risk stratification model was developed which may accurately predict the aneuploidy risk in the ICM [83]. Another study indicated an increase in the rate of segmental aneuploidy on Day-3 [84]. The highest incidence of such aneuploidies are observed at cleavage stage, hypothesizing that first mitotic error divisions may lead to a greater number of mosaic early embryos [84]. The reduction of segmental aneuploidy in the blastocyst stage reveals that the cells in abnormal embryos undergo apoptosis and arrest [84]. The study reported that sites of chromosome breakage are not always random but they depend on specific chromosomal region as observed by the heterochromatic blocks on chromosomes 1, 9 and 16—all being highly affected chromosomes [84].

Despite the undoubtable improvement in techniques employed to identify embryo ploidy status, the detection of mosaicism in embryos remains challenging, as a number of methods such as array-CGH and qPCR are insensitive in detecting low level mosaicism. This may lead to a considerable portion of euploid embryos being inaccurately evaluated as clinically unsuitable and becoming discard material [85,86,87]. Additionally, a comparison between these two methods indicated higher discordant aneuploidy rates for array-CGH [88]. Findings have supported that NGS-based detection methods have the possibility to overpower these limitations and become able to identify precisely mosaicism and segmental aneuploidies [89,90]. Notwithstanding these promising findings, false positive results are still a frustrating matter even with NGS, caused mainly due to the whole genome amplification (WGA) technique, errors in biopsy methodology or poor DNA quality [89,91,92,93,94,95]. In many cases, diagnosis of mosaicism seems to be overestimated owing to both an absence of balanced sensitivity and specificity and strict criteria for mosaicism classification [90,95,96]. Depending on which genetic testing platform has been employed, a false-positive diagnoses incidence may be highly affected [90,96,97]., The predictive value of a biopsy could be also influenced by other factors, including the level of aneuploidy present, the size of the duplication or deletion or even the involved chromosome [90,96]. To complicate matter further, despite the fact that PGT-A has an undoubtedly clinical predictive value in diagnosing chromosomal mosaicism, not all mosaic embryos will fail to implant, but instead may result in healthy live-births [93,94,98,99]. It becomes evident that it may be paradoxical to proceed to biological assumptions while technical uncertainties may influence diagnosis efficacy.

It is undoubtable that the chromosomal complement of the embryo is the major driver that can dictate embryonic fate and the wealth of research on this topic confirms that. The role of aneuploidy and chromosomal abnormalities in embryo development has been thoroughly researched and discussed throughout decades. In more recent years the development of whole genome sequencing techniques enabled enhanced identification of the embryo ploidy status to the extent that on various levels this data is considered accepted rather than experimental knowledge. However, Nonetheless, there are aspects that still present as a mystery and merit further investigation. Mosaicism, and the “secret life” of the embryo towards self-correcting, along with factors affecting mitotic segregation patterns and the cell cycle certainly constitute areas that should be further delineated. Four possible models for embryonic self-correction [30], while one of them was validated by a recent study. The validated mechanism of self-correction has been suggested to entail expelling fragments or abnormal blastomeres. However, this mechanism is not observed in the total cohort of embryos, and may in cases result to the expulsion of euploid blastomeres [100]. It may be possible that the self-correction mechanism may only assist embryos with low level of mosaicism. However, the inherent limitation in confirming embryonic self-correction is the phenomenon of mosaicism itself. Analyzing the whole embryo to fully elucidate the effectiveness of self-correction, may be required, still adequate data employing respective methodology eludes us. Inevitably, further studies are required to elucidate on the mechanism of self-correction and its impact on clinical outcomes where the mosaic embryo is involved.

#### 3.1.6. The Role of the Embryonic Metabolic Profile

During the developmental process, embryos secrete into culture media several substances, which may reveal their developmental potential and may be considered as markers for identifying the optimal embryo to transfer. Caspase-3 is a protein with an established role in apoptosis [101]. It belongs to the executioners group of the caspases family and has a few substrates that are related to DNA fragmentation [102]. Significantly higher levels of caspase-3 in secretomes from arrested embryos were observed when compared with high-quality blastocysts, suggesting that the cause of embryo arrest may be programmed cell death or abnormal cell divisions [103,104,105,106]. Levels of caspase-3 were reported to be higher in low-quality compared with high-quality blastocysts [104].

Additionally, a tendency towards higher levels of Interleukin 6 (IL-6) secretome of blastocyst-formed embryos compared to non-viable embryos has been observed [104]. IL-6 has a fundamental role in preimplantation development and blastocyst hatching similar to Caspase-3, which is known to be involved in the programmed cell death and present in all preimplantation stages [107,108]. Histidine-rich glycoprotein (HRG) is a plasma protein which interacts with thrombospondins preventing them from binding to the receptor CD36 and thereby inhibiting activation of caspases, which may lead to an apoptotic effect [109,110,111]. Furthermore, a major apolipoprotein produced by preimplantation embryos is apolipoprotein A1 (ApoA1), which is associated with high-density lipoprotein (HDL) cholesterol [112,113]. HDL and ApoA1 levels in the follicular fluid are adversely correlated with embryo fragmentation, indicating that oocyte function is affected by HDL prior to embryonic development [114]. Study has shown that levels of ApoA1 are increased in good blastocysts in contrast with poor blastocysts and arrested embryos when their spent media were compared on Day-4 and Day-5 [112]. However, it is worth mentioning that in the observations carried out on Day 3 no ApoA1 mRNA was observed as the embryonic transcriptome is not activated until the 8-cell stage [112,115]. Moreover, the metabolic profile of the preimplantation embryo may be identified by evaluating the levels of pyruvate and glucose uptake and lactate production between fully formed blastocysts and arrested embryos [116]. It is established knowledge that he pyruvate uptake and lactate production in arrested embryos is significantly lower at every developmental stage, while a surge of glycose uptake has been solely observed in viable embryos [116].

A recent study, attempted to analyze the peptides in embryo culture medium and assess their roles in the developmental competency [117]. Findings revealed a total of 201 peptides, originating from 157 precursor proteins, involved in various biological processes, cellular components, and molecular functions namely, immune system process, extracellular region part and binding respectively, differentially expressed in culture medium of embryos that formed blastocysts compared to the medium of embryos that arrested [117]. Furthermore, the study observed a significant increase in peptides derived from precursors HECT And RLD Domain Containing E3 Ubiquitin Protein Ligase 2 (HERC2), PCD16, ATP Binding Cassette Subfamily C Member 8 (ABCC8), and Ubiquitin Protein Ligase E3 Component N-Recognin (UBR4) in the medium with blastocysts [117]. In particular ABCC8, which is suggested to have a vital role in embryo development affecting metabolism of glycose, presented with a 4.03-fold increase in the medium of blastocysts than of non-viable embryos [117,118]. Moreover, UBR4, which promotes mammalian growth participating in the sac vascular development was 2.99-fold higher in blastocyst-formation group [117,119]. Interestingly, the peptide derived from HERC2 was totally absent in culture medium of embryos that failed to form a blastocyst [117]. The HERC2 protein is important for various biological processes namely, cell proliferation [120], DNA repair [121] and apoptosis [122]. HERC2-derived peptide (LGPSVGFDTLRGILISQ) known else as peptide derived from blastocyst culture medium (PDBCM) is possibly secreted by interacting with UBR4 and supports embryo growth [117]. A significant decrease in Cluster of Differentiation 109 (CD109) and Endonuclease/Exonuclease/Phosphatase Family Domain Containing 1 (EEPD1) was observed in blastocyst-containing medium [117]. Another study that also analyzed the different protein levels, indicated significantly higher rates of extracellular matrix metalloproteinase inducer (EMMPRIN) in the culture media of embryos that reached successfully the blastocyst stage than those that arrested [104]. EMMPRIN is a transmembrane glycoprotein receptor and enough crucial for a sufficient implantation and a competent trophoblast differentiation [123].

CDK inhibitors such as p27, which is a member of Cip/Kip inhibitor family, may cause G1 arrest when it is overexpressed in transfected cells [124,125]. The nuclear protein p27 is regulated primarily at the posttranslational level due to protein degradation and has a fundamental role in the pathways of cell differentiation and proliferation [126,127,128]. Significantly higher levels of p27 protein have been observed in the arrested embryos compared to normally developing embryos reaching up to a 2-fold increased expression [128]. Data indicated that transcriptional activation of p27 occurs at the 2-cell stage, thus higher p27 mRNA levels have been observed in the 2- to 4-cell stage [12]. Similarly, low levels of p27 mRNA in healthy embryos may be caused by a failure of transcriptional activation [12]. Besides this, amino acid profile may predict embryo quality through its differentiation across developmental stages [129]. Notwithstanding the fact that mechanisms affecting human preimplantation embryo development are not fully understood many reports attempted to shed light on the role of specific amino acids such as leucine which acts as a signaling molecule and glycine which is involved in osmoregulation process [130,131,132,133]. Amino acid patterns of asparagine, glutamine, arginine, alanine and methionine of Day 2–3 embryos which continued to develop differed from those which arrested [129]. The study reported that in non- viable embryos asparagine, glutamine, arginine, valine, isoleucine and leucine depleted while aspartate, glutamate, glycine and alanine were observed solely on non-viable embryos [129]. This may be attributed to the fact that non- viable embryos are extremely metabolically active [129,134]. The ‘Quiet Embryo Hypothesis’, a characteristic of early embryos introduced a positive correlation between the amount of DNA damage and the overall amino acid turnover [135,136,137,138]. The grounds behind this hypothesis are that a damaged embryo has an increased demand for exogenous nutrients and energy in order to repair DNA damage and optimize protein biosynthesis avoiding an apoptotic effect [135,136,137]. The quiet embryo hypothesis as well as the employment of metabolomics for selecting the optimal embryo to transfer seem promising, however, they have not been validated by large RCTs and meta-analyses. The sole meta-analysis hitherto evaluating metabolomics has reported that no statistically significant difference was observed regarding clinical pregnancy rates [139]. However, the meta-analysis evaluated only four RCTs, with a total sample size of less than 1000 embryos. The small sample, as well as the bias of the included RCTs resulted in low or very-low quality evidence. It is interesting that albeit the “quiet embryo” hypothesis could biologically provide adequate explanation and grounds on efficacious embryo selection, however this scenario has hitherto failed to be verified through clinically effective metabolomic and or proteomic analyses, despite the anticipated promising predictive value. Thus, further research is required either to validate or disprove the quiet embryo hypothesis and the effectiveness of metabolomics in selecting the optimal embryo to transfer.

There are several methods for the evaluation of apoptosis including terminal transferase-mediated DNA end labelling (TUNEL) and annexin V labelling [140,141,142]. Observations of the cellular actin cortex and chromatin indicated that arrested embryos may present with blastomeres with two or more nuclei or even anucleated or with a highly fragmented nucleus [143]. It was revealed that TUNEL assay was positive in around 30% of arrested embryos which exhibited cytoplasmic blebbing and fragmentation, while none TUNEL negative embryos presented with unregular sized or fragmented blastomeres [143]. Moreover, arrested embryos from 2-cells to uncompacted morula stage were annexin V-positive regardless of the rate of their blastomere damage [143]. During the cell cycle, a continuous cooperation between nuclei and cytoplasm is crucial. The nuclear pore complexes (NPC) fuses with nuclear envelope (NE) and supply the channels for bi-directional nucleocytoplasmic trafficking [144,145,146]. A study investigated potential association between the NPC and annulate lamellae (AL), with embryo development [147,148]. Different patterns of AL and NPC assembly were observed in arrested embryos, when evaluated via electron microscopy. Large AL clusters near the pronuclei were observed in zygotes that arrested [148]. Interestingly, arrested embryos presented with similar morphology to viable embryos at the zygote stage, despite the condensed and fragmented DNA. It has been hypothesized that the NE remains intact even in non-viable embryos [148]. Moreover, NPC was highly decreased from the NE in arrested zygotes [148]. The combination of this and DNA fragmentation may reveal an unsuccessful attempt for an apoptotic event [148].

Oxygen consumption has been proposed as a metabolic biomarker, which mirrors the synthesized levels of adenosine triphosphate (ATP) and therefore mitochondrial oxidative phosphorylation process [149]. This biomarker may be of importance when cryopreservation is concerned. The re-formation of the blastocoel immediately following thawing demands high levels of oxygen consumption as the blastocoel cavity is intimately connected with Na/K-ATPase [149,150]. Studies on vitrified-thawed embryos revealed that oxygen consumption rate increased following thawing [149,151]. However, this increase remained significantly higher solely on embryos that recovered rapidly and reached the blastocyst stage compared to those that arrested at each time point [149]. Requirement for more oxygen consumption of embryos in advanced stages is associated with a growing demand for energy in order to compact and form a blastocyst [152,153,154]. Furthermore, mitochondrial cytochrome c oxidase (CCO) activity was observed only at 24 h following thawing, divulging that cryopreservation may affect and cease mitochondrial function [149].

High concentration of reactive oxygen species (ROS) may be associated with embryonic developmental arrest [155,156]. A study indicated positive correlations among ROS levels when evaluated on embryonic Day- 1, 3 and 5 [157]. Interestingly, similar levels were observed on Day-1 in media of normally fertilized zygotes, unfertilized oocytes, and abnormal polyspermic zygotes alike [157]. Moreover, ROS levels on Day-3 and 5 were not significantly related with delayed embryonic cleavage, arrest and blastocyst formation [157]. On the contrary, another study demonstrated that high rates of ROS on Day-1 were responsible for a low fertilization rate, a decreased developmental potential and high fragmentation in embryos delivered with ICSI [158]. In addition, ROS levels on Days 1 and 3 were significantly higher in IVF derived embryos compared to ICSI derived embryos, which may be attributed to different cellular sources of ROS [155,157].

It may be safely concluded that the metabolic profile of an embryo is a non-invasive biomarker that may provide useful information regarding its developmental potential. To further this point, it has been reported that even in the later stages of the preimplantation embryonic development the metabolic profile may assist in predicting the cycle outcome [159,160]. Evaluation of the metabolomic profile along with more established embryo assessment methods may contribute in developing a prediction model that could assist practitioners in selecting the optimal embryo for transfer. Enriching embryo profiling and extending from the genetic to the metabolic and biochemical profile is the key to precision embryology that may lead to customized embryo culture conditions, suitable media composition and embryo individualized conditions in general. Despite the thorough research, the predictive value of the metabolic profile of the embryo has not yet been validated, Hence respective data still requires confirmation prior to considering this as a basis to draw conclusions on. A cost-effective analysis should be performed prior to applying metabolomic profiling in clinical practice, due to the increased financial burden it may weight on both clinics and patients.

### 3.2. The Association between Embryo Morphological Characteristics and Arrested Development

A casual association between multinucleation, delayed cleavage timing and extensive blastomere fragmentation has been established The origins of multinucleation still remain unclear, despite the fact that numerous explanations have been proposed [161]. In fact, more studies should be performed to investigate the possible origin of multinucleation. This is heightened especially as according to the majority of embryo scoring systems, it is categorically described as a poor prognosis factor for the developmental potential of the embryo. Vacuolization is a dynamic process that causes cytoplasmic dysmorphisms in the oocyte and embryo. Homogeneous macrovacuolar formations in oocytes have been reported to result to embryo arrest on day 2 [162]. Before we move to the embryo it is important to highlight the need for further data associating oocyte morphology to arrested development. Vacuoles are generated both spontaneously and by fusion with other already existing vesicles [163,164]. A study revealed that the observation of vacuoles on Day-4 and 5 embryos is associated with developmental arrest as vacuoles impair blastulation [165]. Indeed, on Day-4 a higher number of vacuoles with larger size was observed compared to previous embryonic days [165]. Hypocellular embryos are embryos described as embryos presenting with fewer than the normal number of blastomeres expected. A study revealed that a number of nonviable embryos appeared to be more hypocellular compared to viable embryos with a subsequent lack of compaction on Day-5 [166]. It has been shown that none of these embryos will go on to form a compacted morula on Day-4 or a high-quality blastocyst on the subsequent days [166]. Therefore, hypocellularity may serve as an indicator for embryos to “watch” during culture. A more recent study reported that embryos with fewer than five blastomeres on day 3 present with a significantly lower blastocyst formation rate compared to embryos with five or more blastomeres. The low blastomere number has been associated with fragmentation with potentially a causative relationship observed here [167]. Notably, blastomere fragmentation has been associated with higher levels of caspase-3 and its enhanced activity [103,108]. This is of value considering the role of caspase in apoptosis and perhaps serves to lead the pathway connecting fragmentation, arrested development and apoptosis, a route worth exploring. Interestingly, even if an embryo is deemed unsuitable for embryo transfer on the grounds of arrested development, a number of cells remain viable. This observation merits further investigation as the potential and capacity of these cells along with their respective interactions should be delineated, especially in light of the fact that they have been suggested as a source of stem cells [166,168]. Morphological assessment has been the cornerstone of embryo selection for decades. The associations between specific morphological characteristics and developmental arrest have been thoroughly studied to the extent that they may be viewed as accepted knowledge. However, the associations between embryonic developmental arrest and morphological features merit further investigation in order to reveal the molecular mechanisms and pathways resulting to each phenotype.

### 3.3. Maternal Factors Leading to Preimlantation Embryo Developmental Arrest

A total of 27 studies identified maternal factors as causes of embryonic developmental arrest. Maternal factors describe factors related to the biochemical, genetic, and overall clinical and physical profile of the intended mother contributing the oocyte that forms the embryo studied. In this article the authors have divided maternal factors leading to embryonic arrested development in three distinct categories. Genetic factors (*n* = 15), types of female factor infertility (*n* = 6), as well as biomarkers in the follicular fluid (*n* = 6) have been associated with developmental arrest and are analyzed herein. Following identification of these studies employing systematic review methodology, the authors proceeded with investigating possible associations of infertility etiologies with genetic factors. Subsequently the authors proceeded to explore the connections and mechanisms between the identified genetic factors that were found to be implicated in developmental arrest. This ultimately led to the critical analysis presented herein regarding maternal factors leading to developmental arrest.

#### 3.3.1. The Role of Genetic Factors

Genetic factors represent by nature a multidimensional and vast field of investigation. It appears that a number of gene mutations and genomic alterations may contribute to particular phenotypes. One of the maternal genes associated with embryonic developmental arrest is Peptidyl Arginine Deiminase type-6 (*PADI6*). *PADI6* is a of a family of calcium-dependent cysteine hydrolase enzymes that includes five isotypes (*PADI1-4* and *PADI6*) [169]. Their main role is citrullination, which is a post-translational modification process converting arginine residues to citrulline on protein substrates [170]. *PADI6* is mainly expressed in the oocyte, the ovary, the testis and the early embryo, and its target protein is protamine [169,171]. Thus, *PADI6* is related to different tissue-specific processes, namely *PADI6* presents with a high expression on the oocyte, regulating its activation thus arising to be an important maternal infertility factor [172]. In fact, *PADI6* deficient mice are infertile due to arrested embryonic development at the 2-cell stage [173]. The involvement of the gene, reveals the correlation between oocyte cytoplasmic matrix and ribosomes and emphasizes the contribution of maternal association, with the defective zygote activation which may lead to infertility [174]. These observations are supported by other studies reporting that compound-heterozygous mutations causing lack of *PADI6* expression, result to embryo arrest between the 2-cell and 5-cell stages [173]. *PADI6* has been shown to participate in the subcortical maternal complex and through its recessive variants it has been demonstrated that, its misexpression was related with early embryonic arrest and infertility [174].

Another gene that has been examined regarding its role in embryonic arrest is Tubulin B 8 chain (*TUBB8*). *TUBB8* is a specific β-tubulin isotype, expressed in oocytes and during the first embryo divisions [175]. It is encoded in several tissues among blood, the parathyroid gland and testis and it has a cellular specificity in spermatocytes, early spermatids and spermatogonia. [176]. *TUBB8* is responsible for the expression of the primary beta-tubulin subunit of the oocyte and early embryo. *TUBB8* mutations affect the microtubule behavior and oocyte meiotic spindle assembly, thus they are correlated with oocyte maturation [176]. Therefore, genetic alterations of *TUBB8* are determinant for the process of oocyte maturation and are associated with early embryo developmental arrest [176,177]. It has been reported that, *TUBB8* mutations could be the cause of zygotic arrest in some embryos, by impairing the cell division process. These pathogenetic maternal and zygote patterns, are mainly observed in arrested embryos at the stage of the zygote, as well as at the 8-cell stage [28]. More than 30 mutations linked to embryo arrest have been attributed to TUB88, highlighting the clinical importance of *TUBB8* as a marker of embryo arrest [178].

The subcortical maternal complex (SCMC) is a structure of multiple proteins which is uniquely expressed in early stages of embryo development and mammalian oocytes [179]. This complex is comprised of four key proteins namely, oocyte expressed protein (*OOEP*) which permits embryonic development; nucleotide-binding leucine-rich-repeat (*NLR*) family pyrin domain containing 5 (*NLRP5*) also known as the maternal antigen that embryo requires (*MATER*); transducin-like enhancer of split 6 (*TLE6*) and K-Homology domain containing protein 3 (*KHDC3*) also known as FILIA [180]. The SCMC has a specificity regarding its localization: it is normally situated in the subcortex of murine and human oocytes and preimplantation embryos and is excluded from regions where there is an enhanced cell-to-cell contact in the cleavage-stage embryo [179,181]. The proteins that belong to the SCMC are encoded by maternal effect genes (MEGs) [182,183] and through their encoding maternal transcripts essential for early cleavage events post fertilization are produced [184,185]. They are encoded exclusively in oocytes and early embryos and degrade by the time of the embryonic genome activation, between 4- and 8-cell stage in humans [186], without compensation by embryonic transcription [183]. It appears that genes participating in the subcortical maternal complex play a crucial role in embryo arrest. This could be explained by considering the great importance of the molecular pathways that these genes are involved in; inflammation and apoptosis, oocyte-to-embryo transition, oocyte maturation, first embryo divisions and chromosome recombination. These pathways are crucial steps to determine the sustainability and functionality of the embryo. Employing these genes as diagnostic markers for assessing fertilization failure and embryonic arrest may improve IVF cycle outcomes.

The *NLR* gene family is characterized by a central nucleotide-binding domain, a C-terminal leucine rich repeats (LRR) domain, and an N-terminal effector domain. The N-terminus could be CARD, PYRIN, BIR, AD or X domain, which proves certain homology with CARD or PYRIN [187]. NLR proteins have a significant role in modulating host responses to pathogen-associated molecular patterns and damage-associated molecular patterns [188,189]. NLR family is involved with signaling pathways that regulate mechanisms of cell death [190], inflammation [191], carcinogenesis [192] and autophagy [193]. The NLR family has also been associated with incidents of early embryonic arrest (2- to 7-cell stage), resulting from infertile patients expressing *NLRP2* and *NLRP5* mutations which are illustrated as novel mutant genes [194]. *NLRP7* is a member of SCMC as well and has been demonstrated as a maternal factor involved in inflammation and apoptosis [195]. It appears that single gene mutations of this complex are adequate to determine embryo development and induces an infertility status. *NLRP5* induces primary infertility and Centromere protein H (CENPH) relates to embryonic arrest [196].

*TLE6* is a highly expressed oocyte protein and a main component of SCMC [180]. Mutations in *TLE6* have been related with impaired phosphorylation as *TLE6* is known to be phosphorylated by protein kinase A (PKA) [197]. This affects the binding process with the SCMC complex, providing a potential impact on the phenotypic infertility [181]. Notably, *TLE6* mutations have been associated with early human embryonic lethality [198]. Patients with genetic alterations in *TLE6* experience fertilization failure and early embryonic arrest setting *TLE6* as a possible genetic diagnostic marker [199]. Another significant member of the spectrum of genes responsible for the infertility profiles is Phosphoinositide-Binding Protein (PATL2) which has been correlated with oocyte maturation arrest, fertilization failure, and embryonic developmental arrest [200]. PATL2 is an RNA-binding protein that acts as a translational repressor and has a critical role in the MAPK–PATL2 pathway which is responsible for the regeneration of membranes during cytokinesis [201]. PATL2 plays a major role in oocyte maturation while mutations are linked with infertility both in women and mice. This may be explained considering that PATL2 is expressed in oocytes in the primary follicle stage and is less abundant in the late GV stage [202].

Further investigation of the subcortical maternal complex would provide a more comprehensive knowledge of the molecular pathways and a direct indication of the mechanism. There have been indicated novel biallelic mutations in the SCMC genes *TLE6*, *PADI6* and *KHDC3*L to patients exhibiting embryo arrest presenting from day 2 to day 7 [203]. KH domain-containing 3 like (*KHDC3*L) is encoded in several tissues such as the epididymis, lung, lymphoid tissue, testis and is a subcortical maternal complex member [204].

Variation screening of additional genes has confirmed the impact of zygote arrest 1 (*ZAR1*), a gene contributing to the oocyte-to-embryo transition in mouse and in humans [205,206]. It is an oocyte-specific gene and encodes a protein that is functional in the initiation of embryogenesis [206]. Except from its contribution in early embryo development in oocytes and early embryos, *ZAR1* could also express a determinant impact in the regulation of meiosis and post meiotic differentiation of male and female germ cells. This is also supported by its preserved expression among different species and its regulation may reveal its crucial role in early reproductive procedures. [207]. Through two specific SNPs in *ZAR1* is emerging the association with zygote arrest in humans enhancing the critical role in maternal fertility [208]. Furthermore, participation of meiotic recombination protein 114 (REC114) is required for the formation of double strand breaks (DSBs), a core step towards the initiation of homologous chromosome recombination-.Homozygous mutations cause multiple pronuclei formation and leads to questionable fertilization and early embryonic arrest [209]. The protein expressed by this gene is homologous to the mouse meiotic recombination protein REC114, which is involved in homologous chromosome recombination during meiosis [210]. The encoded protein is conserved in most eukaryotes and was first discovered and characterized in yeast [211].

In the era of precision medicine, the maternal genes entailed in embryonic arrest may be employed during infertility investigation. A detailed description of the gene variations along with sample size and study origin is presented in Table 1. As it may be observed in Table 1, the vast majority of the patients are of East-Asian origin. This may be viewed as a limitation and a possible source of bias, as the other ethnicities are underrepresented. Further studies including a more diverse population are required prior to safely concluding on the effect of specific gene variations in developmental arrest, and prior to identifying this data as accepted knowledge. It may be possible that the genetic profiling of the intended parents may contribute towards a more personalized and patient-oriented approach. Such an approach will upgrade the infertile patient profiling leading to improved prognosis and design of optimal management strategy from stimulation protocols to application of insemination techniques. However, in order for this to be applied in clinical practice, further studies should be conducted to investigate possible genes that may be correlated with embryonic arrested development.

#### 3.3.2. The Role of Follicular Markers

A specific set of mRNAs, luteinizing hormone/choriogonadotropin receptor (LHCGR) mRNA has been identified abundantly in CCs of oocytes leading to embryos associated with live birth rate compared to immature oocytes and oocytes leading to arrested embryos. CCs of oocytes which led to euploid embryos, present with lower hydroxy-Δ-5-steroid dehydrogenase, 3 β- and steroid Δ-isomerase 1 (HSD3B1) mRNA levels compared to those leading to aneuploid blastocysts, and arrested embryos [212]. Genes expression in CCs may differentiate in order to support the development and maturation of the oocyte [33].

In addition, upregulation of prostaglandin-endoperoxide synthase 2 (PTGS2) gene has been associated with lower blastocyst formation rate and poor quality embryos [213]. Follicular fluid metabolites are important parameters for evaluating the relationship between oocyte morphology and developmental competence. Follicular fluid levels of insulin-like growth factors (IGFs), IGF binding proteins (IGFBPs), and pregnancy-associated plasma protein-A (PAPP-A)—a protease for IGFBP-4, may affect the developmental potential of the aspirated oocyte. Embryos that arrested prior to day 2 presented with lower levels of IGF-II, IGFBP-3, and IGFBP-4 in follicular fluid compared to those that reached cleavage stage, whereas the levels of PAPP-A were higher in the arrested group [214]. Another follicular fluid marker that appears to be determinant for embryo development and quality, is the levels of reactive oxygen species (ROS). Levels of ROS in the follicular fluid were associated with poor oocyte grade and fertilization failure leading to low embryo grade and PN arrest [215].

Follicular size has been associated with genetic expression in the CCs, thus serving as a developmental indicator. It has been reported that embryos originating from smaller follicles are more likely to arrest [213]. Follicular growth is mainly determined by FSH and its respective receptor, as well as activin a, and members of the TGF-b superfamily, namely BMP -2, -3b, -4, -6, -7, -15 and GDF-9. As it may be hypothesized, follicular size may relate with other female infertility etiologies in a number of cases. As it may be observed, BMP-15 seems to be an important factor in female infertility affecting embryonic arrest, as it has been observed that its embryonic differential expression may hinder the developmental potential. Follicular biomarkers have been in the spotlight of research for years. However, further studies are required to validate the existing data and perhaps lead to identification and validation of clinically applicable biomarkers.

#### 3.3.3. The Role of Female Infertility Etiology

Apart from, the genetic and molecular causes of embryo arrest, the clinical pathogenetic diagnosis is of substantial value on the interpretation of the underlying female infertility status. Specific etiologies namely, advanced maternal age, endometriosis, poor ovarian response have been associated with developmental arrest.

The incidence of embryonic chromosomal abnormalities is increased due to advanced maternal age. Respectively, women of advanced age are more likely to present with chromosomal abnormalities leading to embryonic arrest and to compromised fertility. The meiotic spindle area and major axis have a fundamental role in proper oocyte development. The expanded size of the meiotic spindle is correlated with embryo developmental arrest prior to reaching the blastocyst stage. Notably, there seems to be an underlining association between major axis of the meiotic spindle and patient age [216]. The age-related decline in female fertility in ART has been well-documented decades [77,217]. This has been attributed to either the diminished receptivity of the older uterus or to quality of the embryos, resulting from aging oocytes. Pioneering work in the 1990′s set the tone and s that maternal age was thought to be correlated with mosaicism, multinucleation, aneuploidy, and anuclear blastomeres all of which hindering the embryo developmental potential [77,217]. However, more recent studies based on NGS have disputed the statement that mosaicism is correlated with maternal age. In fact, recent studies have proposed either a negative correlation [218], or lack of correlation [219,220]. This striking discrepancy in conclusions may be attributed to the fact that maternal age is shown to be positively associated with mosaicism particularly with regard to the X chromosome [221]. It should be noted that sex chromosomes were evaluated in the vast majority of PGT-A cases when the FISH technique was employed. A decrease in the number of euploid embryos has been correlated with advanced maternal age, while the majority of aneuploid embryos may result to developmental arrest [26]. According to studies, the vast majority of trisomies observed due to advanced maternal age may be attributed to errors during the first meiotic division [222]. Another study assessing the effect of maternal age and considering the factors in mosaicism, embryo morphology and chromosomal abnormalities reported extensive mosaicism in arrested embryos. Interestingly no significant correlation of aneuploidy with maternal age was reported when evaluating solely arrested embryos. However, aneuploidy has been shown to be increased with maternal age in non-arrested embryos [77]. Over the years there has been controversy surrounding the extent of association of AMA to aneuploidy and its association to arrested development. Nonetheless, albeit this represents a field of ongoing research generating heated debate, the knowledge that AMA leads to aneuploidy is concrete.

Endometriosis is one of the most crucial idiopathic pathologies that jeopardizes developmental process [223]. An increased incidence of arrested embryos in vitro corresponding to endometriosis parents has been long reported [29]. Endometriosis is an estrogen-dependent chronic inflammatory condition that affect reproductive potential of women resulting to infertility as oocytes retrieved from patients with endometriosis present with abnormal oocyte morphology and lower cytoplasmic mitochondrial content compared to women with other causes of infertility [224,225,226]. Endometriosis is a multi-factorial disease that may bear a genetic background. A recent meta-analysis reported a total of 14 loci that may enhance the risk for endometriosis. The genes included in these loci are involved in WNT signaling, estrogen response, and the actin cytoskeleton and cellular adhesion. However, as the authors mention, genetic predisposition accounts for a limited percentage of cases [227]. On the other hand, a more recent meta-analysis reported that only five polymorphisms in four genes are associated with endometriosis, while a trend was reported for another five polymorphisms in five genes [228]. A consensus point between these two studies is the robust association between WNT4 polymorphisms and endometriosis. WNT signaling has been observed to be involved in folliculogenesis and embryo implantation [229]. Moreover, according to animal studies, WNT signaling has been observed in 8-cell stage mice embryos [230] and in bovine blastocysts [231]. More studies are required to ensure the role of WNT in preimplantation embryonic development. Moreover, miRNAs of the let-7 family have been associated with endometriosis, as their altered expression has been shown to contribute to the pathophysiology of the disease. On the other hand a recent study reported the therapeutic role of the let-7b miRNA [65]. Endometriosis is raising considerable controversy from diagnosis to treatment. Research has shown that several perspectives entailed in this pathology directly impact both oocyte and embryo competence. Ranging from ROS to dysregulation of the immune system, to metalloprotease expression levels and meiotic spindle disruption impacting oocyte and embryo alike, it appears that mapping the molecular mechanism involved will undoubtedly serve the noble intention of precision medicine [232].

Undoubtedly, patients of poor ovarian reserve (POR) are a group of patients presenting with challenges extending to a compromised embryo development. Exploring the impact of this situation on embryo arrest, it has published that POR follicles that lead to arrested embryos present with higher cell free DNA. What compromises DNA integrity and leads to developmentally arrested embryos is the increased necrosis and subsequent diminished steroid hormone levels in the follicular fluid [233]. In order to evaluate the contribution of the morphokinetics of fertilized oocytes from POR patients, studies indicated that more than the half of the embryos present with cleavage irregularities, indicating an estimated high prevalence of developmental arrest [234]. POR is another multifactorial pathology, which may be diagnosed mainly in the context of ART. Genetic factors are among the causes of POR. More specifically a recent meta-analysis reported that mutations in *FMR1*, and *FMR2*, along with polymorphisms in the *BMP-15*, *GDF-9*, in the gene encoding the receptor of FSH (*FSHR*) and in the *NOBOX* gene may present as a cause for POR [235]. The *FMR1* gene is associated with fragile X syndrome in the offspring [236]. *BMP-15* and *GDF-9*, members of the TGF-b superfamily, along with *NOBOX* and have been associated with oogenesis, oocyte growth and maturation [237]. All the above mentioned genes have also been associated with premature ovarian insufficiency [238]. As mentioned above, *BMP-15* mRNA levels in embryos have been positively associated with embryo viability. Furthermore, other candidate genes have also been investigated regarding their role in the pathophysiology of POR, including *BRCA1* and *BRCA2* [235], *IGF-1*, *IGF-2*, along with their respective receptors, *AMH*, *LHCGR* and *GREM1* [239], however robust conclusions cannot yet be reached.

When considering the role of maternal factors leading to embryonic arrest, it becomes clear that besides the directly associated gene variations and follicular factors, infertility etiologies may indirectly influence embryonic development. Advanced maternal age was anticipated to be identified as a major cause of developmental arrest, as it has been associated with aneuploidy. Further to this, endometriosis and POR are two of the most prevalent female infertility etiologies, among females undergoing IVF, according to SART [240]. Their respective genetic background may be indirectly associated with developmental arrest, however further studies are required to directly assess these associations in depth. Should future studies render these finding concrete, then further targeted genetic screening of the intended mother will be of value. This may contribute in optimizing management, from stimulation protocols, to embryo culture decisions aiming to avoid developmental arrest. It should be highlighted that these findings are not accepted knowledge but rather indirect conclusions of the present study towards mapping future research. 

### 3.4. Paternal Factors Leading to Preimplantation Embryo Developmental Arrest

A total of 15 studies investigated paternal factors leading to embryonic arrest. Through the examination of factors involved in developmental arrest, there emerge specific categories revealing contribution of the male factor. More specifically, etiologies include advanced paternal age (*n* = 2), metabolomic profile (*n* = 6), types of male infertility (*n* = 4) and genetic factors (*n* = 3). Following identification of these studies employing systematic review methodology, the authors proceeded with investigating possible associations of infertility etiologies with genetic factors. Subsequently, the authors proceeded to explore the connections and mechanisms between the identified genetic factors that were found to be implicated in developmental arrest. This ultimately led to the critical analysis presented herein regarding paternal factors leading to developmental arrest.

#### 3.4.1. The Role of Genetic Factors

Considering the contribution of genetics in developmental arrest from the perspective of the male factor, it has been observed that genome variations are associated with underlying infertility and compromised embryonic development. A determinant role of estrogen in epigenetic pathways of regulation in male germ line has been reported, proposing that this mechanism in combination with exposure to environmental estrogens may have a critical impact in male fertility [241]. Therefore, analyzing the possible role of RsaI polymorphism of the estrogen receptor-β (ERβ) gene on male population, it was observed that sperm of a specific ERβ RsaI genotype may be related with reduced fertilization and early embryonic developmental potential which may lead to early embryonic developmental arrest [242]. In the same concept, evaluation of postacrosomal WW binding protein (PAWP) in the spermatozoa employed for ICSI, revealed a significant positive correlation between PAWP expression levels and embryonic development [243]. PAWP is a protein important for the stimulation of zygotic development [244]. PAWP is one of the sperm-borne oocyte-activating factors (SOAF) which are essential to stimulate embryonic development [245,246]. These factors are widely conserved among mammals [247]. It has been demonstrated that the microinjection of recombinant PAWP protein is capable of not only stimulating Ca2+ oscillations in mammalian oocytes [248] but also of triggering the release of intracellular Ca2+ in amphibian oocytes [249]. It was also suggested that PAWP is involved in the formation of high-quality spermatozoa [250]. The effect of paternal genetic factors on developmental arrest may be considered as accepted knowledge, however further research is required as it may be possible that other gene variations may affect developmental potential.

#### 3.4.2. The Role of DNA Fragmentation and Chromatin Condensation

DNA fragmentation is a well-examined condition related to male infertility [251]. DNA fragmentation in a semen sample is correlated with diminished fertility and reduces the probabilities of natural conception and intrauterine insemination success [252], as the presence of high levels of DNA damage lead to embryonic arrest. It has been established that sperm DNA integrity affects fertilization rate, developmental arrest, and live birth rates [253]. An increased level of sperm DNA damage is related with lower embryo quality, developmental arrest and decreased pregnancy rates [27]. DNA fragmentation presents with genetic origin as it has been associated with variations in the genes encoding FSH, LH and their respective receptors, as well as with abnormal expression levels of small non-coding RNAs, namely mir-383 and mir-34c [254].

Exploring chromosomal criteria in order to assess paternal influence on the developmental procedure, a correlation between sperm nuclear chromatin condensation and spermatozoa ploidy with embryo development following ICSI has been observed. An increased population of arrested embryos was associated with high levels of sperm nuclear chromatin condensation abnormalities and sperm aneuploidies [255]. Patients with elevated proportion of spermatozoa with single-stranded DNA, presented with an increased number of arrested embryos [256]. Single- stranded DNA has been associated with carcinogenic outcome and cell dysfunctions [257]. Single-stranded DNA is a DNA formation participating in crucial cell processes as recombination, replication and transcription revealing its determinant role on the cellular development [258]. The role of sperm DNA fragmentation and chromatin condensation in the developmental potential of the embryo has been thoroughly researched. Although recent systematic reviews and meta-analyses report a significant correlation between sperm DNA fragmentation and IVF outcomes [259,260], however there is still controversy surrounding the topic [261,262,263,264]. As the meta-analyses feature high heterogeneity, further validation is required.

#### 3.4.3. The Role of Male Infertility Etiologies

Considering the impact of male factor in the developmental process, it should be mentioned that the types of male infertility may affect the developmental process differently. More than half of the embryos originating from azoospermic men arrest between the 2-cell stage and the 6-cell stage [265]. Patients with non-obstructive azoospermia (NOA) subjected to TESE presented with a higher rate of arrested embryos compared to men with obstructive azoospermia (OA) subjected to TESE. Similarly, another study, employing time-lapse microscopy, noticed a declined in the percentage of TESE embryos exhibiting optimal morphokinetical parameters compared with non-TESE embryos [266]. It should be highlighted that azoospermia may be caused by a variety of reasons, including anatomical, iatrogenic or genetic. Numerous genetic disorders may be involved in azoospermia, including microdeletions in the *AZF* genes, variations in genes mainly involved in meiotic arrest [267] and miRNAs, including members of the let7 family, which have been associated with embryonic developmental arrest [268]. It has been reported that besides azoospermia, microdeletions in the *AZF* genes may be responsible for cases of severe oligozoospermia and oligoasthenoteratzoospermia (OAT), while the latter has been associated with autosomal translocations [269]. However, these genetic defects represent only a small proportion of the total population of oligozoospermic or OAT men. Another study, excluding patients with these defects reported that oligoasthenozoospermic and OAT men present with lower cleavage and blastocyst formation rate in comparison to men with a single abnormal parameter or normozoospermic men [270]. Regarding asthenozoospermic men, an increased expression of the let-7b-5p miRNA has been observed. Increased expression of let-7b-5p has been observed in arrested embryos compared to blastocysts [64]. Thus, further profiling may be required in order to evaluate possible monogenic disorders that may lead to male infertility and consequently developmental arrest.

Another type of male infertility, globozoospermia has been related with the outcome of developmental arrest. Globozoospermia is a sub-type of teratozoospermia, where patients present with round-headed spermatozoa and malformation of the acrosome [271]. It has been reported that the contribution of this abnormality may relate to compromised developmental morphokinetics [272]. Globozoospermia is a rare condition of male infertility accounting for less than 0.1% of infertile men [273], and is caused by genetic abnormalities. The most common abnormality is variation in the *DPY19L2* gene, while variations in the *SPATA16*, *PICK1* and *GGN* have also been investigated as causes of globozoospermia [274,275]. Moreover, globozoospermic patients present with high DNA fragmentation index, indicating that there may be more common genetic factors between the two diagnoses, and highlighting the requirement for further investigation.

A study has investigated the potential impact of intracytoplasmic sperm injection (ICSI) along with abnormal semen parameters on developmental potential, and more specifically on blastocyst formation. An increase of arrested embryos in days 3 and 4 was observed when male factor infertility cases where involved, indicating that paternal genomic activation is a determinant factor for embryo development. [276]. It should be highlighted that ICSI was performed on the basis of male factor infertility, whereas in non-male infertility cases conventional IVF was performed. Thus, it cannot be safely concluded if the increase in developmental arrest is caused by ICSI or by male factor infertility. ICSI is an invasive technique, indicated for male factor infertility [277]. Evaluation of ICSI regarding epigenetic alterations has yielded inconclusive results. The minor epigenetic alterations that have been observed to be caused by ICSI [278,279] are thought to be resolved prior to adulthood [280]. However, further longitudinal studies are required in order to evaluate the safety of ICSI.

In men with globozoospermia or other severe defects impairing fertilization potential, the method of artificial oocyte activation (AOA) has been proposed. A study comparing the outcomes of ICSI cycles without AOA to ICSI cycles with AOA using calcium (Ca^2+^) ionophore, observed that the fertilization rate increased from 55% to 67.60%, and slightly less than half of the embryos managed to cleave successfully following application of Ca^2+^ ionophore [281]. It should be noted that only four couples were included in the study. Moreover, It has been hypothesized that AOA with Ca^2+^ may resolve the issue of inadequate signaling events which obstruct oocyte activation and assist in further development [281]. In agreement with the above, another study similarly observed a significantly higher cleavage in zygotes originating from ICSI with calcium ionophore treatment compared with zygotes from conventional ICSI [282]. The study included patients with previous cycles in which at least 85% of the embryos arrested, while the majority of patients presented with cycle cancellation due to complete embryonic arrest. The most impressive observation is the difference in blastocyst formation rate between the AOA group and the conventional ICSI group with, 47.6 versus 5.5%, respectively, by extension resulting in higher incidence of implantation and live births [282]. Despite the promising results, treatment with calcium ionophore remains an IVF add-on. Two meta-analyses have evaluated its efficacy and safety, reporting improved IVF cycle outcome following the AOA [283] and no statistically significant difference in birth defects compared to conventional ICSI [284].

Undoubtedly, age is a common infertility etiology denominator in both women and men [285,286]. In an attempt to isolate the effect of paternal age in embryo development, a study employing donor oocytes noted that despite the fact that no statistically significant difference in fertilization rate of retrieved oocytes, and rate of embryo arrest on cleavage stage was observed, blastocyst formation rate was significantly lower in men over 50 years old [25].

When considering the contribution of male infertility in developmental arrest, different etiologies appear to be hindering embryonic developmental potential. Abnormal chromosomal structure, increased DNA fragmentation and defective chromatin content have been associated with arrested development. Male types of infertility diagnosed according to abnormal semen parameters, may undermine fertilization rate suggesting an impaired developmental process. Despite the fact that limited genes were found to be directly linked with developmental arrest, it may be extrapolated that the genes and sncRNAs causing specific infertility etiologies may exert an indirect effect. However, in order to reach safe and robust conclusions, further research is required to directly associate paternal gene variations or differential expression levels of genes or sncRNAs with developmental arrest of the preimplantation embryo. It should be highlighted that research on the relationship between etiology of male factor infertility and developmental arrest provides verified evidence that could be regarded as accepted knowledge. This evidence pertains mainly to severe male factor infertility. On the other hand, regarding diagnosis of milder male infertility cases, the associations presented herein constitute indirect conclusions that could serve as guidance to mapping future research.

## 4. Discussion

The matter of arrested development has been well documented in literature and albeit there are numerous reports aiming to delineate it, surprisingly hitherto there is no collective, in-depth report on the drivers leading to this phenomenon. It is this gap in literature that fueled conduct of this study, aiming to assist clinical embryologists in better understanding the phenomenon while indicating the focus of future research that should be performed on preimplantation embryo arrested development. This is a unique and -in our opinion-long-overdue contribution to literature that for the first time presents an all-inclusive methodological report subject to the full proof nature of a systematic review on the drivers leading to the preimplantation embryo’s arrested development. What becomes apparent a priori is that the variety and heterogeneity of developmental arrest drivers, along with their inevitable intertwining relationships does not allow for prioritization on the factors playing a more definitive role in preimplantation embryo arrested development.

Novel technologies introduced in the IVF laboratory have allowed for a better understanding of the developmental potential of the preimplantation embryo. The introduction of TLM has enabled continuous monitoring, thus abnormal developmental patterns are easily detectable [30]. The development of whole genome sequencing techniques has enabled not only a more accurate characterization of embryo ploidy but has assisted in providing substantial information regarding the gene variations that may hinder embryonic development [32]. It has been voiced that null-mutations and differential gene expression may be a more frequent cause of embryonic developmental arrest [32]. The early and accurate assessment of the developmental potential of the human embryo is of added value especially in countries where duration of embryo cryopreservation may be extended indefinitely [31].

Developmental arrest is caused by a variety of reasons, that may originate from embryonic or parental factors. A summary of the causes of embryonic arrest is presented in Table 2. It is clear that genetic factors present with the highest impact on embryonic development. This is anticipated as they affect the core identity of the embryo and cannot be circumvented. Chromosomal abnormalities of the embryos either in the form of aneuploidy or translocations detrimentally impair embryo developmental potential. Numerous genes and pathways involved mainly in cycle regulation, DNA repair and structural integrity and maturation of germ cells, may lead to embryonic arrest when differentially expressed or following mutations. On an epigenetic level, hyper- or hypomethylation of specific alleles may impact the possibilities of a successful ART cycle leading to arrested development. The methylation process is crucial for silencing maternal genes and enabling the embryonic genome activation (EGA). EGA is perhaps the most crucial point dictating embryonic fate and reflecting the embryo’s compromised or promising future. The precursor step for embryonic genome activation, is maternal to zygote transition (MZT), a process during which maternal mRNAs are degraded or underexpressed [287]. EGA is believed to be initiated following the 4-cell stage [288]. This may explain the fact that maternal factors mainly cause arrested development that is noted up to the cleavage stage (days 1-3), whereas both paternal and maternal factors may be responsible for embryos that arrest between cavitation of the morula and blastocyst formation (days 4–6). Further studies are required to elucidate the mechanisms of MZT and EGA. Investigation of these mechanisms may assist in further understanding mechanisms involved in the developmental potential of the preimplantation embryos.

Information on the environment and conditions that a gamete and an embryo may be exposed to provide useful insight regarding competence and viability. The numerous metabolites in the follicular fluid, as well as cumulus cells’ gene expression may indicate suitability of the environment of the oocyte, and its potential competence. Similarly, proteins in the seminal plasma may also provide insight on fertilization capability of the spermatozoa. When considering the embryo, the environment is of reciprocal significance, taking into account, the numerous and complex interactions during communication. Undoubtably, the metabolic profile of the embryo may provide a non-invasive biomarker regarding its viability. The conditions under which the embryo develops, ranging from the technique employed for fertilization to culture media and oxygen supplementation, may influence viability and ultimately its suitability for transfer in order to achieve a clinical pregnancy followed by a live-birth. The culture conditions imposed on the embryo may induce possible genetic or epigenetic alterations. Thus, it is of importance to conduct longitudinal studies evaluating the pediatric follow-up data of the offspring originating from IVF.

**Figure 2:**
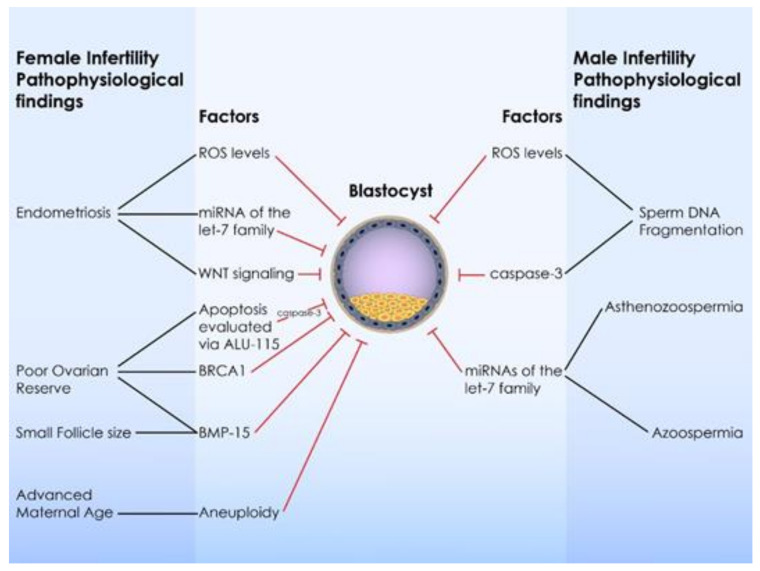
Infertility pathophysiologies and factors leading to developmental arrest of the human preimplantation embryo.

Several infertility etiologies may be indicative of the outcome of developmental arrest. Endometriosis, and poor ovarian response constitute conditions that have been shown to be related to hindering developmental potential of the preimplantation embryos. Similarly, globozoospermia and azoospermia in men present as poor prognostic factors for embryonic viability. Parental age is one of the most common infertility etiologies. Following an exhaustive search according to the systematic methodology employed herein, it became clear that despite the fact that maternal age is extensively studied, little information is provided regarding the influence of paternal age. However, it has been observed that advanced paternal age has been associated with reduced blastocyst formation rate. Further studies should be conducted to enable a more in-depth understanding of the effect of paternal age. Moreover, it may be observed that while a number of maternal originating genetic causes of embryonic arrest have been investigated, the search yielded a limited number of results regarding the paternal genetic profile. It may be timely and essential to evaluate male infertility beyond the scope of a semen analysis and the common anatomical etiologies to a deeper genomic wide profiling of the intended father. While this may not be employed in clinical practice directly, research may reveal possible biomarkers which in turn may be indicative of the outcome. Perhaps the fact that a diagnosis of male infertility can be circumvented through employment on techniques such as ICSI to ensure fertilization, may explain the lack of incentive behind aspiring to treat some forms of male infertility. Enhancing the research field of male infertility may consequently lead to development of different approaches resulting to enhanced IVF cycle outcomes in terms of both safety and efficacy. A graphic illustration presenting parental infertility etiologies and suggested factors that may affect embryonic developmental potential is presented in Figure 2. As it may be observed in Figure 2, the vast majority of male and female infertility etiologies present with at least one factor that has been associated with developmental arrest. However, this is an indirect cause. Further studies delineating on the infertility etiologies and developmental arrest are required.

The effects of DNA fragmentation have been studied in both males and females presenting as an apoptotic marker. In males it may been evaluated in the form of sperm DNA fragmentation. In women on the other hand identification of this marker is more complicated. Studies have investigated ALU fragments in follicular fluid of POR patients. It has been observed that the levels of these apoptotic factors may hinder the IVF cycle outcome. Apoptotic events in embryos hinder developmental potential. However, there is still lack of an all-inclusive study. According to the observations enabled by this systematic review it may be of importance to evaluate the possible apoptotic mechanisms by which parental DNA fragmentation may affect subsequent embryo development. Implementation of this non-invasive biomarker during the infertility evaluation may be of prognostic value. Investigation of the apoptotic pathways and the factors that may affect them, may provide personalized treatment for the infertile couple, which could in turn improve IVF outcomes.

One would expect that oocyte morphology would be associated with embryonic developmental arrest. However, the majority of studies present with great heterogeneity and a relevant systematic review, performed a decade ago, was unable to reach robust conclusions [289]. More recent studies have presented an association between oocyte morphology and embryonic developmental potential [290,291], without however presenting a causative association. It may be possible that the effect of the oocyte in embryonic development may be indirect, affecting primarily insemination outcome prior to the developmental fate of the embryo [292]. More studies on the genetic aspect of oocyte morphology may be crucial in investigating its effect on embryonic developmental potential.

**Figure 3:**
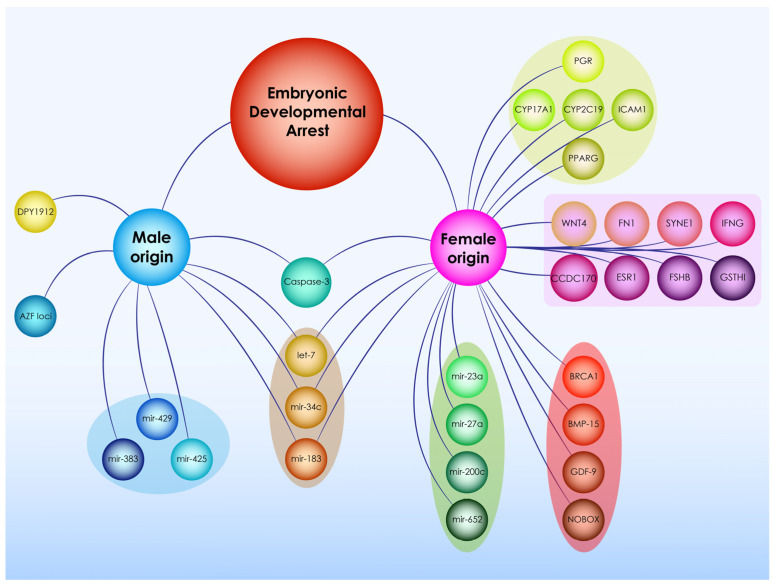
Mapping future research areas in arrested development of the human preimplantation embryo.

Results yielded herein following a systematic review methodology indicate the need for further studies on unveiling the molecular backbone of embryonic arrest, accounting for various sources from intrinsic genetic factors to the clinical profile of the intended parents. Future research should focus on genetic and epigenetic alterations that may influence developmental potential. It may be of interest to perform whole-genome sequencing in sibling embryos and evaluate possible loci that may be responsible for arrest. However, this type of research design may not yield specific results and may not be cost-effective. A targeted research evaluating variations as well as their expression levels in genes presenting with a correlation to a known infertility pathophysiology may yield more conclusive results. The fact that infertility pathophysiology seems to share a common ground with developmental arrest strengthens the proposal that these pathways should be considered for further research. This type of research design should be considered as a first step in investigating the origins of developmental arrest. Further to this, it has been established that miRNA expression levels are associated with infertility pathophysiologies [66,292,293]. A recent study in mice reported 10 miRNAs as potential candidates associated with developmental arrest [294]. A number of them have been similarly associated with infertility in humans. It may be of interest to elucidate on their role in infertility pathophysiology and developmental arrest in the preimplantation embryo. A graphic illustration presenting a map for future research, including the above-mentioned factors is presented in Figure 3. Another aspect that may merit serious investigation is senescence and its role and developmental disorders of the human embryo in the IVF laboratory. One could hypothesize that developmental arrest is a process similar to cellular senescence [295]. It is possible that similar pathways may be involved in both phenomena, as they may be sharing more common ground than we are aware of. Investigating morphokinetic findings that correspond to events of senescence in the human IVF preimplantation embryo may represent a highly promising area of research. Evaluation of senescence markers, namely lipofuscin [296], in preimplantation embryos may assist in further understanding embryonic developmental arrest.

Understanding the drivers of developmental arrest may assist in future attempts to enhance blastocyst formation rates, thus indirectly improving clinical pregnancy and live-birth rates. The promise of precision medicine relies on the concept that identification of the genetic or molecular causes of rare phenotypes will lead to adjusting respective treatments accordingly, to address the particular needs of each individual patient. In the case of arrested development of the preimplantation embryo the hypothesis is formed that understanding the molecular drivers behind the phenomenon can ascertain optimal treatment. However, hitherto, only a limited number of studies have focused on addressing this issue and improve developmental arrest rates. Recently a study on animal models, and more specifically mice, reported that the novel and promising technique of maternal spindle transfer may enable overcoming this phenomenon of in cases where developmental arrest is triggered by defective ooplasm [297]. Since this a proof-of-concept study, further research is required prior to validating this technique in humans and ascertain safe and effective employment.

It should be mentioned that embryonic arrested development is not a primary outcome for all the studies in the field. Thus, it may not be directly mentioned in the studies. This may present as a limitation for the present systematic review. Moreover, conclusions pertaining to female infertility etiology and mainly to male infertility etiology are indirect, thus the results of the present study should be interpreted with a degree of caution. Hence, it is of importance to conduct further studies directly investigating the parental molecular pathways that lead to developmental arrest. It should be noted that along with the said intrinsic factors leading to arrested development, extrinsic factors present as an equal contributor. Albeit this review focuses strictly on the intrinsic factors, it should be stated that both elements can impact synergistically embryo potential development. When contemplating arrested development extrinsic factors ranging from laboratory conditions, techniques, operating protocols and the role of the practitioner should be taken into account. It has been well established that environmental conditions and culture media are of paramount importance, as poor culture conditions may restrict embryo developmental potential subjecting the embryo to stress that is unequivocally related to a compromised developmental outcome [298,299]. It is imperative to research techniques and culture conditions in ART leading to developmental arrest. The insemination techniques, culture media, oxygen levels, cryopreservation and IVF add-ons employed in an IVF laboratory may epigenetically influence the embryo, thus altering its developmental potential [300]. It has been voiced that ICSI may differentiate the methylation patterns as evaluated in newborn blood [301,302]. Further to this, cryopreservation of oocytes or embryos has been considered as a cause of epigenetic modifications [303,304]. Regarding the embryo biopsy data on animal models epigenetic alterations are showcased [305]. However, further studies in humans are required, especially on blastocyst biopsy, which has been demonstrated to be more effective compared to cleavage stage biopsy [306]. Moreover, optimal culture media and conditions are equally important. Addition of various factors including amino acids, proteins and vitamins into the culture media may have a key role throughout preimplantation development affecting embryo competence [307,308]. Similarly identification of optimal settings and subsequent application of numerous laboratory variables, namely temperature, oxygen, humidity and carbon dioxide is an absolute requirement as they may compromise embryonic development [307]. It should be mentioned however, that the majority of epigenetic alterations in IVF offsprings are considered to be resolved till adulthood [280]. Further research in the field is crucial to assist in understanding and managing developmental arrest, enabling personalized treatment approach.

This systematic review provides the basis for further research regarding embryonic developmental arrest. The goal of the study is to assist in understanding and set the fundamentals for further attempts to control and possibly avoid developmental arrest. Investigating the factors that may lead to developmental arrest will raise awareness on one of the most crucial and complicated processes in assisted reproduction that is still underexplored. Profiling of the intended parents along with elucidating on the factors that may cause genetic or epigenetic alterations during embryonic development may be equally vital to proper embryo profiling in the era where precision embryology is the future. Successful identification of the reasons causing developmental arrest may assist research focusing on development of improved technologies and optimizing IVF treatment. Albeit current options in addressing arrested development challenge clinical practice, this direction remains a noble goal fueling formation of hypotheses and development of novel approaches that merit validation. This would ultimately ascertain a greater number of embryos available for embryo transfer and higher percentage of live-births.

## Figures and Tables

**Table 1:** Characteristics of the studies on the effect of maternal gene variations on developmental arrest.

Study ID	GENE	Variations	Study Origin	Sample Size	
Tian	*ZAR1*	rs117545505 and rs17609740	China	47 women with cleavage failure	Controls: 93 women undergoing IVF/ICSI and 188 women with natural conception

Sha	*TUBB8*	NP	China	66 women	
Chen	*TUBB8*	28 variants	China	30 families	
Yuan	*TUBB8*	c.322G>A	China	1 woman and 178 controls	
Wang	*REC114*	c.546 + 5G>A	China	2 families	
Zheng	*PADI6*	c.1521dupC, c.A1117C and c.C1708T	China	2 women	
Quian	*PADI6*	c.1793A>G and c.2045 G>A,	China	1 family	
Wang 2018	*PADI6*	c.866C>T, c.1895C>T and c.1124dupT	China	2 families and 80 infertile controls	
Xu	*PADI6*	c.2009_2010del c.633T>A c.1618G>A c.970C>T	China	2 out of 36 women	
Mu	*NLRP2*	c.1961C>A, c.773T>C, c.2254C>T, c.525G>C, c.2544A>T, c.662C>T, c.1847A>T, c.1469C>T	China	5 families	
Mu	*NLRP5*	c.292C>T, c.2081C>T, c.866G>A, c.3320C>T	China	2 families	
Wu	*PATL2*	p.V260M, p.Q300*, p.T425P, p.D293Y p.N239Tfs*9 p.R75Vfs*21	China	5 families	
Wang 2018	*TLE6*	c.1133delC	China, however the family was from Ukraine	1 family and 80 infertile controls	
Wang 2018	*KHDC3L*	c.44delA	China	1 family and 80 infertile controls	

**Table 2:** Factors affecting developmental arrest.

Factor	Type	Effect	Possible Underlying Infertility Pathophysiology		Screening
Cyclins, Cyclin-dependent kinases and their inhibitors (CCNA2, CDK1, CDK2, CDK4, p27)	Protein expression levels	Embryo	NP		Proteomic analysis
DNMT3B, HDAC1, TET3 and YY1	Gene variation	Embryo	NP		Genetic analysis
ATG5, CUL3, USP11 and USP2	Gene variation	Embryo	NP		Genetic analysis
TERF1, ERCC1 and XRCC6	Gene variation	Embryo	NP		Genetic analysis
BRCA1	Gene variation	Both	POR		Genetic analysis
ITPR1	Gene variation	Maternal	NP		Genetic analysis
4977-bp mtDNA deletion	Copy numbers	Parental	NP		Genetic analysis
HO-1	Gene variation	Parental	NP		Genetic analysis
H19DMR	ICRs	Parental	NP		Transcriptomic analysis
Nanog and Oct4 transcription factors	Protein expression levels	Embryo	NP		Methylome analysis
piRNA	sncRNA	Embryo	NP		Transcriptomic analysis
miRNA mainly of the let-7 family	sncRNA	Both	In females, miRNAs have been associated with endometriosis		Transcriptomic analysis
			In males, miRNAs have been associated with azoospermia and asthenozoospermia		
Aneuploidy, mosaicism	Chromosomal abnormalities	Embryo	Advanced maternal age		Genetic analysis
Caspase-3	Protein expression levels	Both	sperm DNA fragmentation		Proteomic analysis
IL-6	Protein expression levels	Embryo	NP		Proteomic analysis
HRG	Protein expression levels	Embryo	NP		Proteomic analysis
HDL and ApoA1	Protein expression levels	Embryo	NP		Proteomic analysis
ABCC8	Gene expression levels	Embryo	NP		Transcriptomic analysis
UBR4	Gene expression levels	Embryo	NP		Transcriptomic analysis
HERC2	Gene expression levels	Embryo	NP		Transcriptomic analysis
EMMPRIN	Gene expression levels	Embryo	NP		Transcriptomic analysis
Oxygen consumption	Metabolic biomarker	Embryo	NP		Metabolomic analysis
ROS	Metabolic biomarker	Both	ROS in the follicular fluid is related to poor oocyte quality and fertilization failure.		Metabolomic analysis
			Seminal ROS levels are associated with sperm DNA fragmentation.		
*PADI6*	Gene variation	Maternal	oocyte activation failure		Genetic analysis in parents
*TUBB8*	Gene variation	Both	oocyte meiotic spindle assembly		Genetic analysis in parents
NLRP7, *TLE6*, *KHDC3*, *OOEP*	Gene variation	Maternal	Formation of subcortical maternal complex		Genetic analysis
NRLP5	Gene variation	Maternal	Primary infertility		Genetic analysis
PATL2	Gene variation	Maternal	NP		Genetic analysis
*ZAR1*	Gene variation	Both	Affects MZT		Genetic analysis
REC114	Protein expression levels	Maternal	NP		Proteomic analysis in parents
IGF-1, -2 and their respective receptors	Protein or mRNA expression levels	Maternal	POR		Proteomic or transcriptomic analysis in parents
IGFBPs, and PAPP-A	Protein expression levels	Maternal	NP		Proteomic analysis in parents
FSH, LH and their receptors	Gene variation or Expression levels	Parental	In females: follicular growth	In males: DNA fragmentation, azoospermia	Genetic or Proteomic analysis in parents
Activin a	Protein expression levels	Parental	Follicular growth		Proteomic analysis in parents
WNT4	Gene variation	Maternal	Endometriosis		Genetic analysis in parents
FMR1, GDF-9 and NOBOX	Gene variation	Maternal	POR		Genetic analysis in parents
*BMP-15*	Gene variation	Both	POR and follicular growth		Genetic analysis in parents
PAWP	Protein expression levels	Paternal	Oocyte activation		Proteomic analysis in parents
*AZF*	Gene variation	Paternal	Azoospermia, oligozoospermia and oligoasthenoteratzoospermia		Genetic analysis in parents
DPY19L2, SPATA16, PICK1 and GGN	Gene variation	Paternal	Globozoospermia		Genetic analysis in parents

NP: Not provided; Both: Affecting both the embryo and at least one parent.

## Data Availability

Not applicable.
